# Flow cytometric detection of vancomycin-resistant* Enterococcus faecium* in urine using fluorescently labelled enterocin K1

**DOI:** 10.1038/s41598-023-38114-9

**Published:** 2023-07-06

**Authors:** Thomas F. Oftedal, Dzung B. Diep

**Affiliations:** Faculty of Chemistry, Biotechnology and Food Science, Norwegian University of Life Sciences, Ås, Norway

**Keywords:** Fluorescent dyes, Fluorescent proteins, Microbiology techniques, Urinary tract infection

## Abstract

A urinary tract infection (UTI) occurs when bacteria enter and multiply in the urinary system. The infection is most often caused by enteric bacteria that normally live in the gut, which include *Enterococcus faecium*. Without antibiotic treatment, UTIs can progress to life-threatening septic shock. Early diagnosis and identification of the pathogen will reduce antibiotic use and improve patient outcomes. In this work, we develop and optimize a cost-effective and rapid (< 40 min) method for detecting *E. faecium* in urine. The method uses a fluorescently labelled bacteriocin enterocin K1 (FITC-EntK1) that binds specifically to *E. faecium* and is then detected using a conventional flow cytometer. Using this detection assay, urine containing *E. faecium* was identified by an increase in the fluorescent signals by 25–73-fold (median fluorescence intensity) compared to control samples containing *Escherichia coli* or *Staphylococcus aureus*. The method presented in this work is a proof of concept showing the potential of bacteriocins to act as specific probes for the detection of specific bacteria, such as pathogens, in biological samples.

## Introduction

Urinary tract infections (UTIs) are among the most common infections in humans, and account for significant health-care costs and morbidity^1–3^. Women are predominantly affected by UTIs with 13% of women self-reporting having a UTI compared with 3% of men (data from NHANES III, 1988–1994)^4^. UTIs are most commonly caused by bacteria entering the urethra, and usually involve bacteria of the gut microbiota^5^. A UTI is an infection in any part of the urinary tract, such as the bladder, ureters, urethra, or kidneys, but occurs most commonly in the bladder (cystitis), which can progress to pyelonephritis (infection of the kidney)^5^. In pregnant women UTIs are associated with preterm birth and reduced birth weight of the infant^6,7^. If left untreated, UTIs can lead to complications such as kidney stones or systemic bloodstream infections^8,9^. The laboratory diagnostic criterium for UTIs is the presence of at least one bacterial species with a total count ≥ 10^5^ CFU/ml which is determined via urine culture of midstream urine, a diagnostic procedure that typically takes 24–48 h^10^. Because urine culture is slow, determination of the causative microorganism and its antibiotic resistance profile is rarely obtained prior to management of the infection^11^. Consequently, most clinical guidelines currently recommend the diagnosis and management of uncomplicated UTIs based solely on symptoms^11^. In some cases, up to 90% of patients with urinary symptoms receive antibiotics, often without obtaining a urine culture^11^. A faster diagnostic procedure for UTIs would increase positive health outcomes in patients and reduce the unnecessary use of antibiotics^12,13^.

In recent years, new methods have been proposed for faster diagnosis of UTIs, such as special-purpose flow cytometers and direct biotyping from urine using matrix assisted laser desorption/ionization time-of-flight mass spectrometry (MALDI-TOF MS)^14–17^. A major drawback of MALDI-TOF MS is the cost associated with the acquisition of the instrumentation and the proprietary software and databases necessary for its clinical use^18^. Special-purpose flow cytometers such as the Sysmex urinalysis devices are more affordable, easy to use, and claims to rule out potential UTIs within minutes^19^. The Sysmex devices rely on a dedicated mixing chamber where all bacteria are stained with a fluorescent nucleic acid binding dye, which is necessary for detection^15^. Antimicrobial peptides (AMPs) and antibiotics have been explored for the labeling and detection of pathogenic bacteria. Labeled ubiquicidin (29–41) was shown to localize to the sites of infection by *Pseudomonas aeruginosa* or *Staphylococcus aureus* in mice^20,21^. Similarly, fluorescently labelled vancomycin was shown to detect infections by *S. aureus* in a mouse myositis model^22^. Using Cy5-labeled cecropin P1, detection of *Escherichia coli* O157:H7 was enhanced tenfold compared to antibody-based detection^23^. AMPs produced by bacteria are known as bacteriocins, which resemble AMPs in many aspects. However, bacteriocins have much higher potency and a narrow spectrum of activity, typically being active only towards species closely related to the producer^24^. The narrow targeting of many bacteriocins is due to specific receptor molecules exploited by these peptides to target cells^25–27^. Bacteriocins show high potency and specificity towards many species of bacteria, including those implicated in UTIs^28–30^. However, the use of bacteriocins for detection remains largely unexplored. Many bacteriocins, especially those that are unmodified, can easily be synthesized commercially with fluorescent labels.

Bacteriocins are a heterogeneous group of ribosomally synthesized antimicrobial peptides produced by virtually all bacterial species^31^. Although bacteriocins are typically only active against species closely related to the producer, there are bacteriocins with broad-spectrum activity^32–34^. Bacteriocins are particularly interesting because of their high specificity and high potency against antibiotic-resistant strains^35^. Members of the LsbB family of bacteriocins include enterocin EJ97 (EntEJ97), enterocin K1 (EntK1), lactococcal small bacteriocin B (LsbB), enterocin Q (EntQ) and the engineered hybrid bacteriocin H1^36–38^. All members are small (30–44 amino acids), leaderless, unmodified, and exploit the same membrane-bound site-2 metalloprotease RseP as a receptor for its antimicrobial activity^38–40^. The C-terminal tail of these bacteriocins is thought to be important for receptor interaction^41^. While enterocin EJ97 displays a broader inhibition spectrum including *E. faecium* and *E. faecalis*, EntK1 and LsbB have a much narrower inhibition spectrum. LsbB is only active against strains of *L. lactis,* while EntK1 mostly toward *E. faecium*, including both nosocomial and vancomycin-resistant (VRE) strains^38^. The target specificity of the bacteriocins is primarily due to subtle sequence differences in RseP between species^38–40^. The small and unmodified nature of the LsbB family of bacteriocins makes them ideal for synthetic production and chemical modifications, that can be used to develop them into useful tools for therapeutic and diagnostic applications.

The aim of this study was to develop the narrow spectrum bacteriocin EntK1 into a molecular probe for cost- and time-effective detection of *E. faecium*. The procedure involves a binding step that allows the fluorescent peptide to bind to target cells, followed by detection using a conventional flow cytometer. We further validated the procedure with urine samples to simulate UTIs. We believe that the potential of a fast and species-specific detection method offered by these peptides would reduce the unnecessary use of antibiotics.

## Results

To enable detection of the bacteriocin EntK1, the peptide was chemically synthesized with a FITC fluorescent label conjugated to the N-terminus. FITC is a small (389 Da) and widely used fluorophore with excitation and emission maxima typically measured at 494 nm and 518 nm, respectively^42^. The fluorophore was chosen due to its relatively small size (380 Da) compared to EntK1 (4564 Da) and conjugated to the N-terminus to avoid interfering with the bacteriocin-receptor interaction^25,41^. The minimum inhibitory concentration (MIC) of the labelled EntK1 (FITC-EntK1) was at nanomolar concentrations against *E. faecium* LMGT 3104 (a VRE strain; also designated LMG 20705)^33^. The low MIC of the modified peptide indicates that it is still relatively potent, although the potency was reduced about fourfold compared to the non-modified EntK1 (156 nM for FITC-EntK1 compared to 39 nM for EntK1). A concentration slightly above the MIC_90_ of FITC-EntK1 (0.2 µM) was chosen for further binding experiments. In this work, the term “binding” will be used to describe any measurable association of FITC-EntK1 with cells.

**Figure 1:**
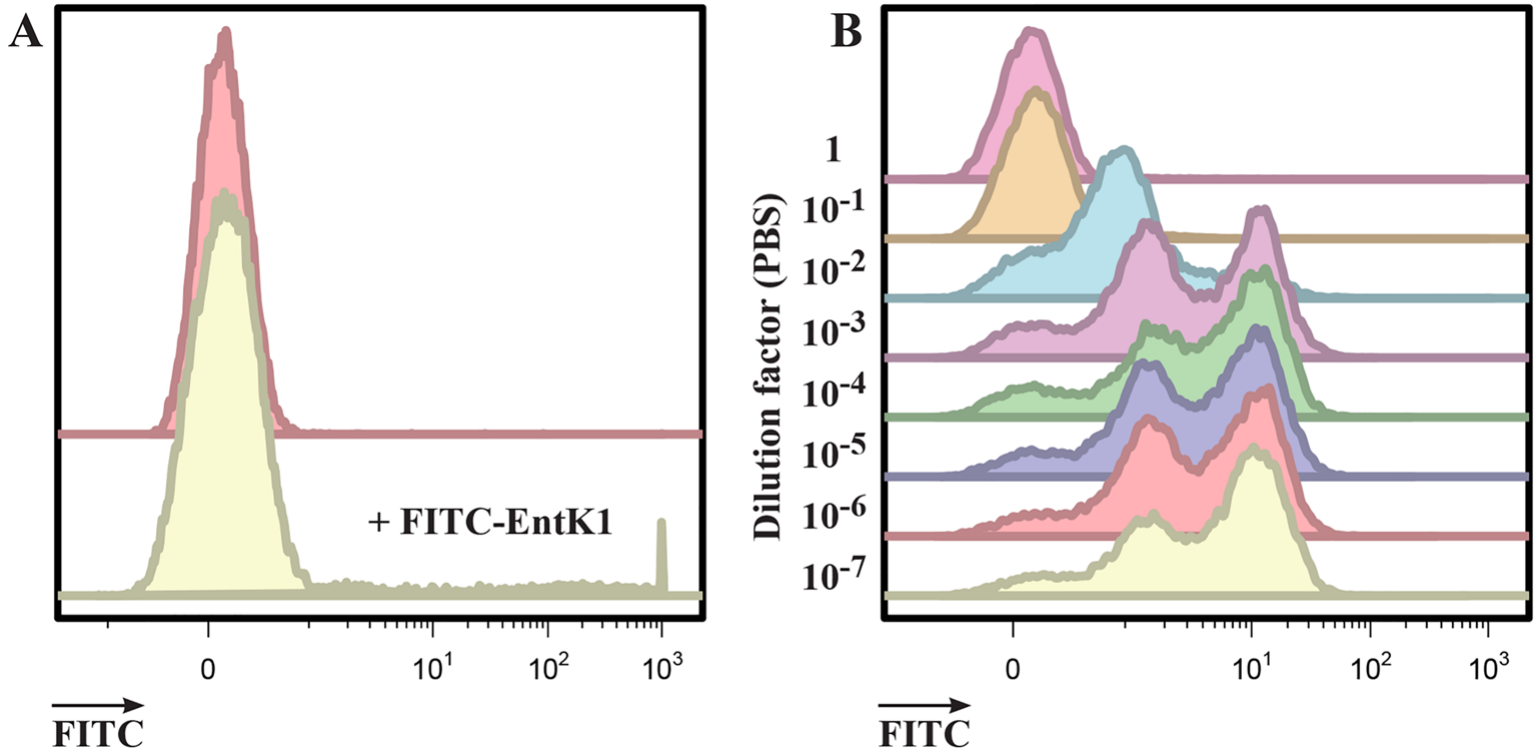
*E. faecium* at approximately 10^5^ CFU/ml incubated for 2 h in PBS. (**A**) With and without 1 µM FITC-EntK1, as indicated. (**B**) Binding performed in PBS and a tenfold serial dilution of PBS in pure water from undiluted 1X PBS (top) to 10^–7^ times diluted (bottom).

Initial attempts at measuring the binding of FITC-EntK1 to *E. faecium* in physiological buffers (such as PBS) using flow cytometry were not successful. The increase in the fluorescence signal in samples with added FITC-EntK1 was negligible compared to unstained controls, even when the concentration of FITC-EntK1 was increased to 1 µM and the incubation time increased to 2 h (see Fig. 1A). The failure to detect cells with bound FITC-EntK1 could be due to cell death and lysis, however, the mode of action appears to be non-lytic, and no morphological changes of the cells were apparent even after 2 h (see Fig. S1). In addition, the number of events measured by the flow cytometer from samples with FITC-EntK1 were comparable to unexposed controls. However, when using diluted buffers during the binding step, the fluorescence intensity of *E. faecium* increased (Fig. 1B).

**Figure 2:**
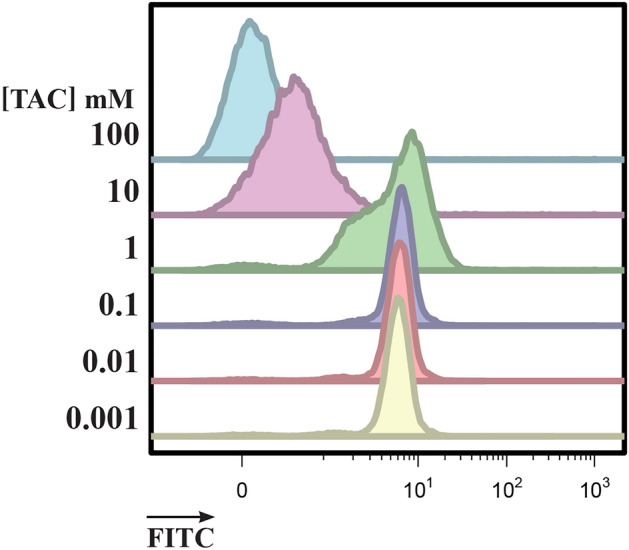
Binding assay of FITC-EntK1 to *E. faecium* (10^5^ CFU/ml). The assay was performed in varying concentrations from 100 to 0.001 mM triammonium citrate buffer (TAC) with a 15 min binding step.

Although the fluorescence of *E. faecium* increased in dilute PBS, the binding appeared to be inefficient as the distribution had two peaks (bimodal) with a long tail, resulting in reduced median fluorescence intensity values (MFI). To investigate if other buffers could improve binding, a large selection of buffers were tested with 0.2 µM FITC-EntK1 at varying incubation times and ionic strengths. Best results were obtained with the citrate-based buffer triammonium citrate pH 6.8 (TAC) at a concentration of 0.1 mM (Fig. 2). Interestingly, in this buffer, the maximum fluorescence was obtained after only 15 min of incubation, and samples with longer incubation times showed no or only a negligible further increase (see Fig. S2).

**Figure 3:**
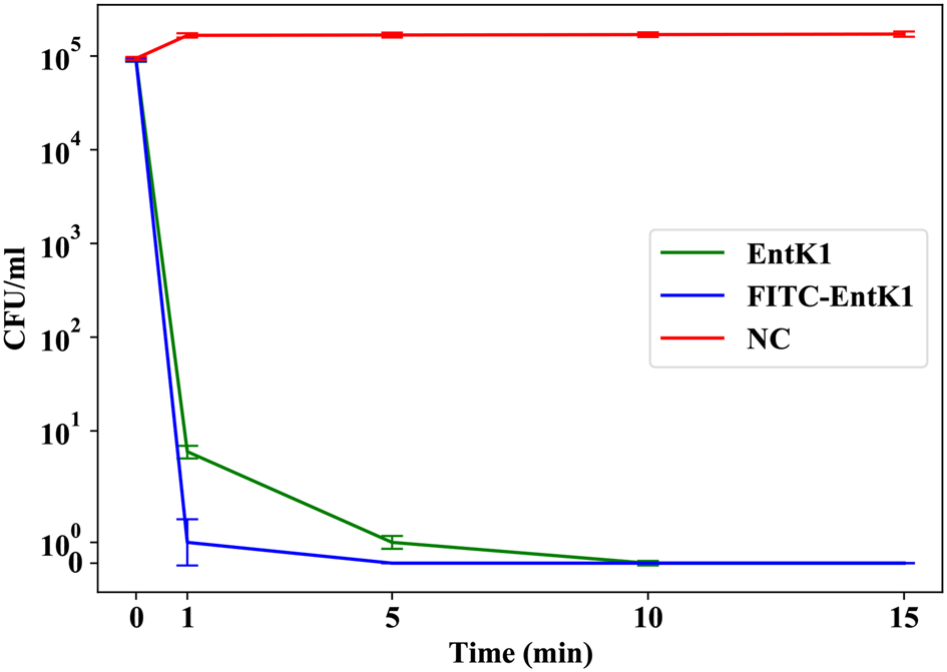
Killing kinetics assay. Number of viable cells in colony-forming units (CFU) in 5 ml of 0.1 mM TAC buffer following the addition of EntK1 (green line) or FITC-EntK1 (blue line) to 0.2 µM. A negative control with no added antimicrobial is shown in red (NC). Error bars are ± SE (standard error). The figure was generated using Python 3.8.8 with a symlog y-axis (linear in the range −5 to 5).

The short incubation time of 15 min was confirmed by a killing kinetics assay showing that EntK1 and FITC-EntK1 kill target cells rapidly in 0.1 mM TAC buffer. As seen in Fig. 3, exposure to both EntK1 and FITC-EntK1 at 0.2 µM resulted in a 4-log reduction in viable cells after one minute, with a complete reduction of viable cells after 15 min of exposure to the bacteriocins. The binding buffer alone showed no reduction in cell viability.

**Figure 4:**
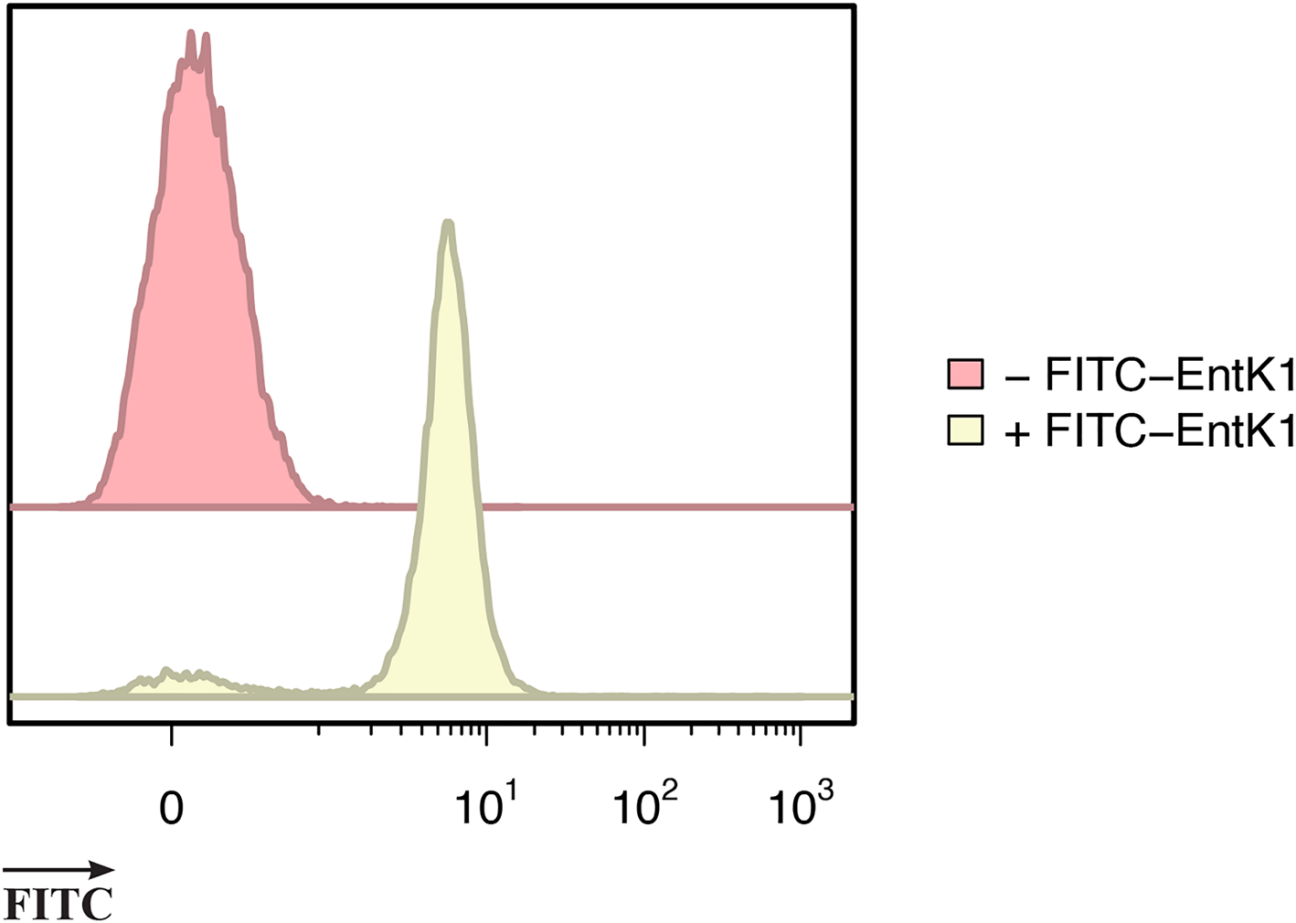
FITC-EntK1 binds to *E. faecium* from urine. Fluorescence of unstained *E. faecium* LMGT 3104 from urine (red), fluorescence following a 15 min incubation with FITC-EntK1 (yellow). Representative figure from twelve independent experiments.

Next we attempted to demonstrate the binding of FITC-EntK1 to *E. faecium* originating from urine. To do this, a clinical case of UTI was simulated by adding 10^5^ CFU/ml of *E. faecium* LMGT 3104 to urine samples. The measured binding of FITC-EntK1 to *E. faecium* directly in urine was small with a high sample-to-sample variance, likely due to the relatively high and varying salt content. To remove the effect of solutes in urine on binding, a one-step enrichment of bacteria was first performed by centrifugation. Using flow cytometry, samples containing *E. faecium* showed a 48–59-fold increase in the median fluorescence intensity (MFI) following a 15-min incubation with 0.2 µM FITC-EntK1 in 0.1 mM TAC buffer (see Fig. 4). A similar fold-increase was observed for all biological replicates. Samples containing *E. faecium* with no added FITC-EntK1 had a mean MFI value of 0.1 (n = 12, SD = 0.027, *p*-value = 0.000028). The reproducibility of the method made it possible to distinguish urine samples containing *E. faecium* (i.e., from an infected individual) from a healthy control.

**Figure 5:**
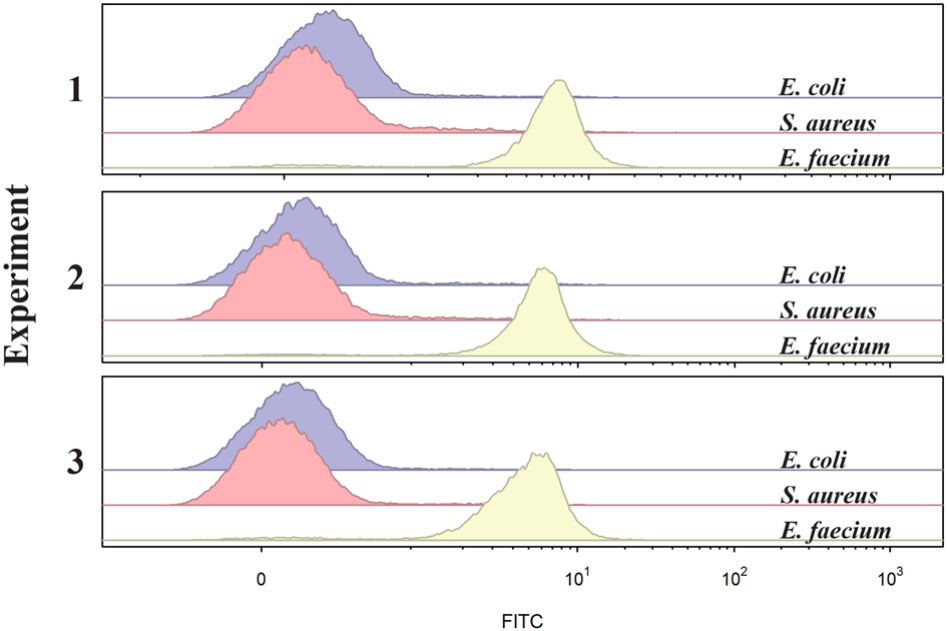
Fluorescence (FITC; 525/50 nm) obtained from urine samples containing *S. aureus*, *E. coli*, and *E. faecium*. Samples containing *E. faecium* show a positive shift in fluorescence (three independent experiments).

To further investigate if the observed binding was specific to *E. faecium* or if FITC-EntK1 in 0.1 mM TAC buffer would bind unspecifically to any bacteria present in the sample, two species also implicated in UTIs namely *E. coli* and *S. aureus* were included in the assays. Both strains were confirmed to be insensitive to EntK1 and FITC-EntK1 (MIC_90_ > 200 µM). As shown in Fig. 5, only urine samples containing *E. faecium* showed a shift in fluorescence intensity.

Samples with *E. coli* or *S. aureus* showed similar fluorescence values to controls with no added FITC-EntK1 (corresponding to background levels of fluorescence). The increase in MFI for *E. faecium* relative to *S. aureus* or *E. coli* was 25–73-fold and reproducible in all assays performed (see Table 1). Density plots of SSC-A and FSC-A for these experiments are presented in Fig. S3.

**Table 1:** Summary of three independent detection assays.

Replicate	CFU/ml*	MFI
*E. faecium*		
1	9.3 × 10^4^	5.88
2	9.6 × 10^4^	5.80
3	8.8 × 10^4^	4.87
*S. aureus*		
1	1.17 × 10^5^	0.10
2	9 × 10^4^	0.11
3	1.03 × 10^4^	0.08
*E. coli*		
1	1.07 × 10^5^	0.19
2	1.06 × 10^5^	0.17
3	1.15 × 10^5^	0.13

Urine samples containing *E. faecium*, *E. coli* and *S. aureus* (three biological replicates). Total bacteria count determined for each urine sample used in the assay is shown in CFU/ml.

*Mean of three technical replicates, rounded to the nearest thousand.

**Figure 6:**
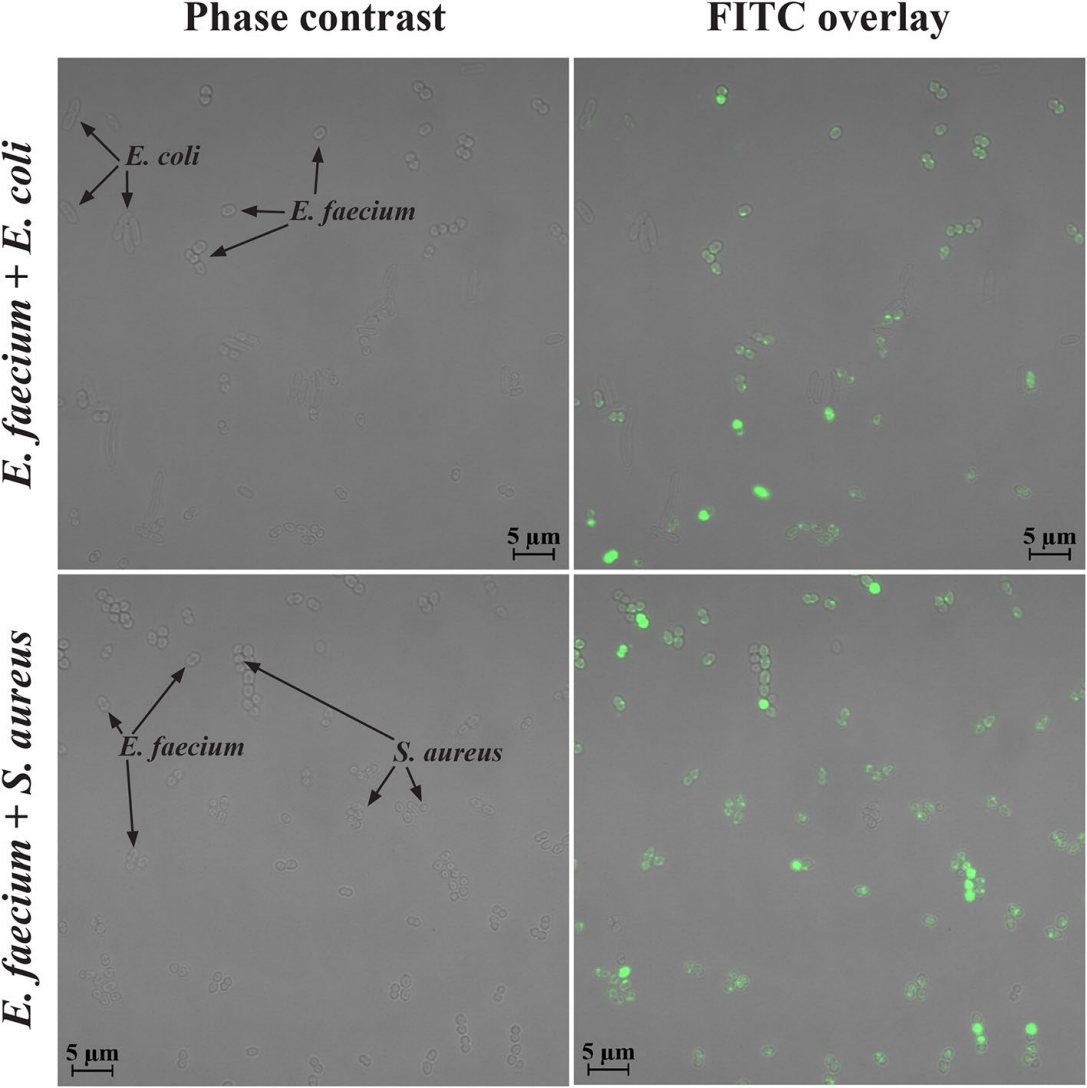
Fluorescence microscopy of mixed cultures. *E. faecium* mixed with *S. aureus* (**A**), and *E. faecium* mixed with *E. coli* (**B**). Images were taken following a 15-min incubation in 0.1 mM TAC buffer with 0.2 µM FITC-EntK1, cells were mixed at equal numbers. Overlay of fluorescence and phase-contrast (transmitted light) channels.

During a UTI, a second microorganism might be present at a high number together with the causative agent, typically with at least one of them present at 10^5^ CFU/mL or more. The simultaneous presence of another microorganism could influence and interfere with the binding of FITC-EntK1 to *E. faecium*. To test this, 10^5^ CFU/ml of *E. faecium* was pre-mixed with 10^5^ CFU/ml of *E. coli* or *S. aureus*. The binding of FITC-EntK1 to *E. faecium* in the presence of another species was examined by confocal laser scanning microscopy (CLSM), which allowed us to distinguish each species based on differences in morphology (see Fig. 6).

Cells with a morphology consistent with *E. faecium* (diplococci) exhibited a visible fluorescence signal. While a lesser signal or no signal was apparent for *S. aureus,* which are predominantly in chains or clusters as single cells (cocci, spherical), while *E. coli* are rod-shaped cells.

**Figure 7:**
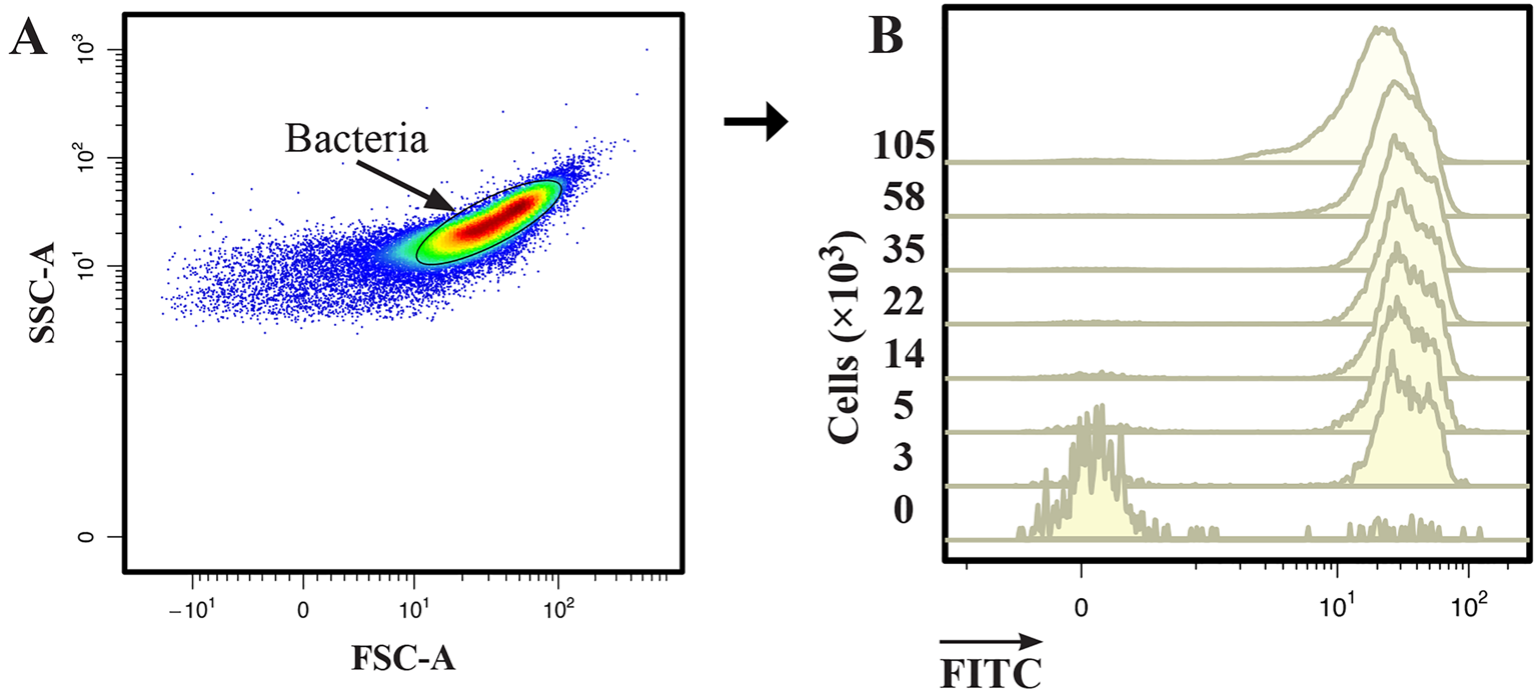
Limit of detection. A gate was constructed for the sample with the highest count of *E. faecium*, and fluorescence was measured on events within the gate (**A**). Fluorescence signal from urine samples containing the indicated number of cells in CFU/ml (**B**). Representative figures from three independent experiments.

Although many laboratories use a bacterial count ≥ 10^5^ CFU/ml as a diagnostic criterion for UTIs, many laboratories have opted to use a lower colony count of 10^3^–10^4^ CFU/ml^43,44^. The higher threshold has been shown to miss many relevant infections, as healthy urine should otherwise appear sterile by commonly used cultivation techniques. A lower threshold will detect more cases of UTIs and allow for earlier intervention. To determine the ability of the presented method to detect *E. faecium* in urine samples at the lower threshold of 10^3^–10^4^ CFU/ml, a serial dilution of cells from ~ 10^5^ to 3 × 10^3^ CFU/ml was prepared in urine. A sample with no added bacteria was included as a comparison. Because of the high relative proportion of noise at low cell counts, control samples of urine without added *E. faecium* and with *E. faecium* only (without added FITC-EntK1) were used to determine the light scattering characteristics of *E. faecium* bacteria. Based on these controls, a gating strategy was constructed to capture *E. faecium* and reduce noise (Fig. 7A).

As shown in Fig. 7B, urine samples inoculated with *E. faecium* were clearly distinguishable from the control even when present at only 3 × 10^3^ CFU/ml**.** The MFI was 30.1 for the lowest cell count tested, compared to 0.11 for the control with no added cells. Fluorescence and gating data are presented in Table S1 and density plots in Fig. S4.

## Materials and methods

### Bacteriocin stock preparation

Enterocin K1 (EntK1) and FITC-EntK1 were synthesized by Pepmic Co., Ltd. (Suzhou, China) with > 95% purity. The FITC fluorescent label was conjugated to the N-terminal via a 6-aminohexanoic acid linker. Both peptides were solubilized in MilliQ water to a stock concentration of 200 µM for use in all assays.

### Minimum inhibitory concentration

Twofold dilutions of EntK1 and FITC-EntK1 in BHI were prepared in 96-well microtiter plates to a volume of 100 μl per well. Each well was then inoculated with 100 μl of a 25-fold diluted overnight culture of *E. faecium* LMGT 3104 (50-fold final dilution). After incubation at 37 °C for 6 h, the turbidity was measured by a spectrophotometer SPECTROstar Nano reader (BMG Labtech) at 600 nm. The MIC_90_ was defined as the concentration of bacteriocin necessary to inhibit growth by 90% or more in 200 µl of culture (having a turbidity equal to 10% or less of a positive control with no antimicrobial).

### Killing kinetics assay

A culture of *E. faecium* LMGT 3104 was grown overnight at 37 °C in Brain Heart Infusion broth (BHI). The culture was diluted in BHI to the desired cell count (using a standard curve of turbidity/OD_600_ to CFU per ml) before being added to 5 ml of binding buffer (0.1 mM tri-ammonium citrate, 0.25 M sucrose, pH 6.5) to approximately 10^5^ CFU/ml. Actual bacterial counts in each suspension were determined in all assays by plate counting. Briefly, samples were immediately diluted 100-fold in sterile saline (0.9% NaCl) and 0.1 ml of the dilution spread on BHI agar plates. Bacteriocins EntK1 and FITC-EntK1 was added to 0.2 µM final concentration and samples were taken for plate counting as described above at 1, 5, 10 and 15 min. A control with no added antimicrobial was also included to assess any potential antimicrobial effect of the binding buffer. The assay was performed in triplicate, and the data presented is the mean of all assays with the corresponding sample standard deviation (SD).

### Sample preparation

Urine was sampled from healthy laboratory staff (30 ml per sample) and artificially inoculated with approximately 10^5^ CFU/ml of *E. faecium* LMGT 3104, *E. coli* TG1, or *S. aureus* RN4220 as described above. For the limit of detection experiments, a serial dilution of *E. faecium* was prepared in sterile saline (0.9% NaCl) before being added to the urine sample. After adding bacteria to the samples, the actual bacterial counts in all samples were determined by serial dilution in sterile saline and plate counting (three technical replicates per biological replicate). Cells were collected by centrifugation (7500 g, 5 min) and resuspended in binding buffer (0.1 mM tri-ammonium citrate, 0.25 M sucrose, pH 6.5) containing 0.2 µM FITC-EntK1. Samples were incubated for 15 min on a Multi Bio RS-24 rotator (BioSan, Riga, Latvia) at room temperature for binding. Following the binding step, cells were filtered through a 20 µm cell-strainer (EASYstrainer small, Greiner) and washed once in sterile-filtered phosphate-buffered saline (PBS; 137 mM NaCl, 2.6 mM KCl, 10 mM Na_2_HPO_4_, 1.8 mM KH_2_PO_4_, pH 7.2) by centrifugation as described above, then resuspended thoroughly in 0.5 ml of PBS by vortexing for 5–10 s. The suspension (25 µl) was directly measured by flow cytometry.

### Flow cytometry

All samples were analyzed by flow cytometry using a MACSQuant Analyzer (Miltenyi Biotec, Bergisch Gladbach, Germany). Events were recorded using a low flow rate (25 µL/min) using the green 488 nm laser for excitation (25 mW laser power) and emission detector B1 (525/50 nm filter) with a detector voltage of 400 V. A trigger threshold was set to 3 using side scattered light (SSC-H; 370 V detector voltage) to reduce excess noise in the measurements. Except for the limit of detection experiments, all flow cytometry data was ungated, and all recorded events were included in the calculations. Statistical comparison was performed using the Mann–Whitney U Test implemented in R. The grating strategy used for the limit of detection experiments is provided in Fig. 7A. Data and figures were prepared using the CytoExploreR package (v 1.1.0) for the R programming language (v 4.1.1).

### Microscopy

Urine samples (30 ml) were inoculated with mixed cultures at approximately 5 × 10^4^ CFU/ml of *E. faecium* LMGT 3104, *S. aureus* RN4220 or *E. coli* TG1. The cells were stained with the FITC-labeled EntK1 as described for the sample preparation above. After the washing step, the cells were resuspended in 25 μl of PBS and then spotted on a microscopy slide overlayed with 2% low-melting agarose in PBS to immobilize the cells. Phase-contrast images and FITC fluorescence images were obtained using a confocal laser scanning microscope (LSM700, Axio Observer.Z1, Zeiss, Germany) equipped with an EC Plan-Neofluoar 100x/1.3 objective. Fluorescence was detected with excitation using the 488 nm laser line and measuring emission at wavelengths above 510 nm. Images were processed with ZEN 2012 software.

## Discussion

In this study, we show that the bacteriocin FITC-EntK1 can function as a molecular probe that preferentially binds to or associates with *E. faecium*. The detection assay presented in this work allowed us to positively identify urine samples with 10^5^ CFU/ml of *E. faecium* present. The assay is both rapid and appears to be species-specific, which could enable early and targeted intervention in a clinical setting. Additionally, a clear shift in fluorescence was observed for urine samples containing as few as 3 × 10^3^ CFU/ml compared to healthy controls, which is below the lowest clinical threshold proposed for the diagnosis of UTIs^45,46^. Although the prevalence of UTIs caused by *E. faecium* is relatively low (~ 2%)^47^, the concept of using bacteriocins as probes for detection and diagnosis is largely unexplored. There exists a great diversity of bacteriocins that target various pathogenic species in a specific receptor-mediated manner that could be developed for detection, as demonstrated in this work for EntK1. Bacteriocins active against the most prevalent urinary pathogens have been characterized, such as colicins and microcins against *E. coli*, klebicins against *Klebsiella,* and pyocins against *Pseudomonas*^48–50^. Many bacteriocins targeting Gram-negative bacteria are large proteins (40–70 kDa) and therefore likely impractical as probes. However, it seems plausible that only the smaller receptor-binding domain of such bacteriocins would bind with high affinity to the receptor and could therefore function as probes.

In our study, careful optimization of the binding conditions was necessary to demonstrate the binding of FITC-EntK1 to *E. faecium* populations. Buffers with high ionic strength, such as PBS, showed only a negligible difference in the fluorescent signals produced by *E. faecium* with or without FITC-EntK1. In contrast, all species tested showed binding to FITC-EntK1 in all non-ionic solutions, likely due to unspecific electrostatic interactions with the cell surface. By gradually decreasing the ionic strength of all buffers tested, the binding of FITC-EntK1 to *E. faecium* increased (see Fig. 2). Presumably, the ions in solution shield or neutralize the charges on the cell surface and bacteriocin, thereby reducing electrostatic interactions between the bacteriocin and cells. The effect of solutes on bacteriocin adsorption to cells suggests that defined conditions will be necessary for a reliable detection system.

The low binding measured between FITC-EntK1 and cells in physiological buffers is consistent with previous literature showing reduced sensitivity to bacteriocins in solutions of increasing ionic strength, which is assumed to lower the affinity of the peptides to the cell surface^51–53^. Interactions of bacteriocins such as EntK1 with the cell surface is initially believed to be dominated by electrostatic interactions^54^. Bacteriocins predominantly contain an excess of positively charged amino acids (EntK1 has a net charge of 5 at pH 7 and an isoelectric point at pH 10.17), and the bacterial cell surface possesses a net negative electrostatic charge due to phosphoryl and carboxylate groups^54,55^.

Detection of pathogenic bacteria directly from biological fluids without the need for a separation step would reduce the protocol time. However, we believe bacterial separation and enrichment from bodily fluids other than blood could be performed in minutes by simple filtration and/or centrifugation techniques, as demonstrated for urine. All binding experiments in this work used urine sampled from healthy individuals, urine from infected individuals often contains traces of blood and/or neutrophils (pyuria) that could interfere with the detection assay in a clinical setting. Most eukaryotic cells should not pass the 20 µm filter used in the assay protocol, however, further work is needed to assess the method using urine from infected individuals. In the absence of fluorescent labels, conventional flow cytometers are poor at small-particle detection (< 3 µm) such as bacteria (0.5–2 µm), which appear indistinguishable from noise (e.g., inherent electrical noise, internal reflections, stray light, or dust and debris in the buffers) when analyzed by light scatter. However, by selectively staining bacteria with fluorescent bacteriocins such as FITC-EntK1, bacteria can be detected with sufficient sensitivity.

This work presents a proof of concept of using bacteriocins with specific activity as probes for the detection of target bacteria. The detection assay developed in this work for EntK1 shows good sensitivity and specificity, positively identifying urine samples containing the clinical threshold of 10^5^ CFU/ml of *E. faecium*. The detection assay could likely be further developed and optimized for other bacteriocins and for other clinically important bodily fluids such as cerebrospinal, synovial, ascitic, or amniotic fluids. We foresee a role of bacteriocins in the design and development of diagnostic kits and methods, providing rapid and specific identification of their target bacteria. However, further work is needed to establish the potential and broader applicability of the proof of concept presented in this work.

## Supplementary Information

Supplementary Information.

## Data Availability

The data underlying the results presented in the study are available upon request to T.F.O at thof@nmbu.no.

## References

[1] Foxman B (2010). The epidemiology of urinary tract infection. Nat Rev Urol.

[2] Abbo LM, Hooton TM (2014). Antimicrobial stewardship and urinary tract infections. Antibiotics.

[3] Medina M, Castillo-Pino E (2019). An introduction to the epidemiology and burden of urinary tract infections. Ther Adv Urol.

[4] Foxman B, Barlow R, D’Arcy H, Gillespie B, Sobel JD (2000). Urinary tract infection: Self-reported incidence and associated costs. Ann. Epidemiol..

[5] Lee JBL, Neild GH (2007). Urinary tract infection. Medicine.

[6] Glaser AP, Schaeffer AJ (2015). Urinary tract infection and bacteriuria in pregnancy. Urol. Clin..

[7] Chu CM, Lowder JL (2018). Diagnosis and treatment of urinary tract infections across age groups. Am. J. Obstet. Gynecol..

[8] Ripa, F. *et al.* Association of kidney stones and recurrent UTIs: The chicken and egg situation. A systematic review of literature. *Curr Urol Rep* **23**, 165–174 (2022). 10.1007/s11934-022-01103-y PMC9492590 35877059

[9] Gharbi M (2019). Antibiotic management of urinary tract infection in elderly patients in primary care and its association with bloodstream infections and all cause mortality: population based cohort study. BMJ.

[10] Rowe TA, Juthani-Mehta M (2014). Diagnosis and management of urinary tract infection in older adults. Infect. Dis. Clin. North Am..

[11] Fraile Navarro D, Sullivan F, Azcoaga-Lorenzo A, Hernandez Santiago V (2020). Point-of-care tests for urinary tract infections: protocol for a systematic review and meta-analysis of diagnostic test accuracy. BMJ Open.

[12] McIsaac WJ, Hunchak CL (2011). Overestimation error and unnecessary antibiotic prescriptions for acute cystitis in adult women. Med Decis Making.

[13] Kumar A (2009). Initiation of inappropriate antimicrobial therapy results in a fivefold reduction of survival in human septic shock. Chest.

[14] Zboromyrska Y (2016). Development of a new protocol for rapid bacterial identification and susceptibility testing directly from urine samples. Clin. Microbiol. Infect..

[15] Rubio E (2019). Evaluation of flow cytometry for the detection of bacteria in biological fluids. PLoS ONE.

[16] Ferreira L (2010). Direct identification of urinary tract pathogens from urine samples by matrix-assisted laser desorption ionization-time of flight mass spectrometry. J. Clin. Microbiol..

[17] Íñigo M (2016). Direct identification of urinary tract pathogens from urine samples, combining urine screening methods and matrix-assisted laser desorption ionization-time of flight mass spectrometry. J. Clin. Microbiol..

[18] Singhal, N., Kumar, M., Kanaujia, P. K. & Virdi, J. S. MALDI-TOF mass spectrometry: an emerging technology for microbial identification and diagnosis. *Front. Microbio.* **6**, (2015). 10.3389/fmicb.2015.00791 PMC4525378 26300860

[19] De Rosa R (2010). Evaluation of the Sysmex UF1000i flow cytometer for ruling out bacterial urinary tract infection. Clin. Chim. Acta.

[20] Liu C, Gu Y (2013). Noninvasive optical imaging of *staphylococcus aureus* infection in vivo using an antimicrobial peptide fragment based near-infrared fluorescent probes. J. Innov. Opt. Health Sci..

[21] Lupetti A, Welling MM, Pauwels EK, Nibbering PH (2003). Radiolabelled antimicrobial peptides for infection detection. Lancet. Infect. Dis.

[22] van Oosten M (2013). Real-time in vivo imaging of invasive- and biomaterial-associated bacterial infections using fluorescently labelled vancomycin. Nat Commun.

[23] Arcidiacono S, Pivarnik P, Mello CM, Senecal A (2008). Cy5 labeled antimicrobial peptides for enhanced detection of *Escherichia coli* O157:H7. Biosens. Bioelectron..

[24] Nissen-Meyer J, Nes IF (1997). Ribosomally synthesized antimicrobial peptides: their function, structure, biogenesis, and mechanism of action. Arch Microbiol.

[25] Kristensen SS (2022). The extracellular domain of site-2-metalloprotease RseP is important for sensitivity to bacteriocin EntK1. J. Biol. Chem..

[26] Kjos M, Salehian Z, Nes IF, Diep DB (2010). An extracellular loop of the mannose phosphotransferase system component IIC is responsible for specific targeting by class IIa bacteriocins. J. Bacteriol..

[27] Cotter PD (2014). An ‘Upp’-turn in bacteriocin receptor identification. Mol. Microbiol..

[28] Huang F (2021). Bacteriocins: potential for human health. Oxid. Med. Cell. Longev..

[29] Choi, G.-H., Holzapfel, W. H., & Todorov, S. D. Diversity of the bacteriocins, their classification and potential applications in combat of antibiotic resistant and clinically relevant pathogens. *Crit. Rev. Microbiol.* **0**, 1–20 (2022). 10.1080/1040841X.2022.2090227 35731254

[30] Telhig, S., Ben Said, L., Zirah, S., Fliss, I. & Rebuffat, S. Bacteriocins to Thwart bacterial resistance in gram negative bacteria. *Front. Microbiol.* **11**, (2020). 10.3389/fmicb.2020.586433 PMC7680869 33240239

[31] Riley MA, Wertz JE (2002). Bacteriocins: Evolution, ecology, and application. Annu. Rev. Microbiol..

[32] McAuliffe O (1998). Lacticin 3147, a broad-spectrum bacteriocin which selectively dissipates the membrane potential. Appl. Environ. Microbiol..

[33] Rosenbergová Z (2022). Identification of a novel two-peptide lantibiotic from *Vagococcus fluvialis*. Microbiol. Spectrum.

[34] Ovchinnikov KV (2016). Novel group of leaderless multipeptide bacteriocins from gram-positive bacteria. Appl. Environ. Microbiol..

[35] Soltani, S. *et al.* Bacteriocins as a new generation of antimicrobials: toxicity aspects and regulations. *FEMS Microbiol. Rev.* **45**, fuaa039 (2021). 10.1093/femsre/fuaa039 PMC7794045 32876664

[36] Cintas LM (2000). Biochemical and genetic evidence that *Enterococcus faecium* L50 produces enterocins L50A and L50B, the sec-dependent enterocin P, and a novel bacteriocin secreted without an N-terminal extension termed enterocin Q. J. Bacteriol..

[37] Gajic O (2003). Novel mechanism of bacteriocin secretion and immunity carried out by lactococcal multidrug resistance proteins*. J. Biol. Chem..

[38] Ovchinnikov, K. V. *et al.* The leaderless bacteriocin enterocin K1 is highly potent against *Enterococcus faecium*: A study on structure, target spectrum and receptor. *Front. Microbiol.* **8** (2017). 10.3389/fmicb.2017.00774 PMC5413573 28515717

[39] Kranjec C (2021). A bacteriocin-based treatment option for *Staphylococcus haemolyticus* biofilms. Sci Rep.

[40] Miljkovic M (2016). LsbB bacteriocin interacts with the third transmembrane domain of the YvjB receptor. Appl. Environ. Microbiol..

[41] Ovchinnikov KV (2014). Defining the structure and receptor binding domain of the leaderless bacteriocin LsbB *. J. Biol. Chem..

[42] Holm T (2006). Studying the uptake of cell-penetrating peptides. Nat Protoc.

[43] Wilson ML, Gaido L (2004). Laboratory diagnosis of urinary tract infections in adult patients. Clin. Infect. Dis..

[44] Schmiemann G, Kniehl E, Gebhardt K, Matejczyk MM, Hummers-Pradier E (2010). The diagnosis of urinary tract infection. Dtsch Arztebl Int.

[45] Roberts KB, Wald ER (2018). The diagnosis of UTI: Colony count criteria revisited. Pediatrics.

[46] Primack W, Bukowski T, Sutherland R, Gravens-Mueller L, Carpenter M (2017). What urinary colony count indicates a urinary tract infection in children?. J Pediatr.

[47] Serretiello E (2021). Trend of bacterial uropathogens and their susceptibility pattern: Study of single academic high-volume Center in Italy (2015–2019). Int J Microbiol.

[48] Michel-Briand Y, Baysse C (2002). The pyocins of *Pseudomonas aeruginosa*. Biochimie.

[49] Denkovskienė E (2019). Broad and efficient control of Klebsiella pathogens by peptidoglycan-degrading and pore-forming bacteriocins klebicins. Sci Rep.

[50] Duquesne S, Destoumieux-Garzón D, Peduzzi J, Rebuffat S (2007). Microcins, gene-encoded antibacterial peptides from enterobacteria. Nat. Prod. Rep..

[51] Bhunia, A. k., Johnson, M. c., Ray, B. & Kalchayanand, N. Mode of action of pediocin AcH from *Pediococcus acidilactici* H on sensitive bacterial strains. *J. Appl. Bacteriol.* **70**, 25–33 (1991).

[52] Atrih A, Rekhif N, Moir AJG, Lebrihi A, Lefebvre G (2001). Mode of action, purification and amino acid sequence of plantaricin C19, an anti-Listeria bacteriocin produced by *Lactobacillus plantarum* C19. Int. J. Food Microbiol..

[53] Kazazic M, Nissen-Meyer J, Fimland G (2002). Mutational analysis of the role of charged residues in target-cell binding, potency and specificity of the pediocin-like bacteriocin sakacin P. Microbiology.

[54] Perez, R. H., Zendo, T. & Sonomoto, K. Circular and leaderless bacteriocins: Biosynthesis, mode of action, applications, and prospects. *Front. Microbiol.* **9** (2018). 10.3389/fmicb.2018.02085 PMC6131525 30233551

[55] Chen Y, Ludescher RD, Montville TJ (1997). Electrostatic interactions, but not the YGNGV consensus motif, govern the binding of pediocin PA-1 and its fragments to phospholipid vesicles. Appl. Environ. Microbiol..

