# Supplementary material for: Flow cytometric detection of vancomycin-resistant Enterococcus faecium in urine using fluorescently labelled enterocin K1
Source: Sci Rep. 2023 Jul 6;13:10930. doi: 10.1038/s41598-023-38114-9 (PMC10325980; doi:10.1038/s41598-023-38114-9)
Supplement: Supplementary file 1 — Supplementary Information. [file 41598_2023_38114_MOESM1_ESM.pdf]

Flow cytometric detection of vancomycin-resistant *Enterococcus faecium* in urine using fluorescently labelled enterocin K1

Thomas F. Oftedal[1,*] and Dzung B. Diep[1,†]

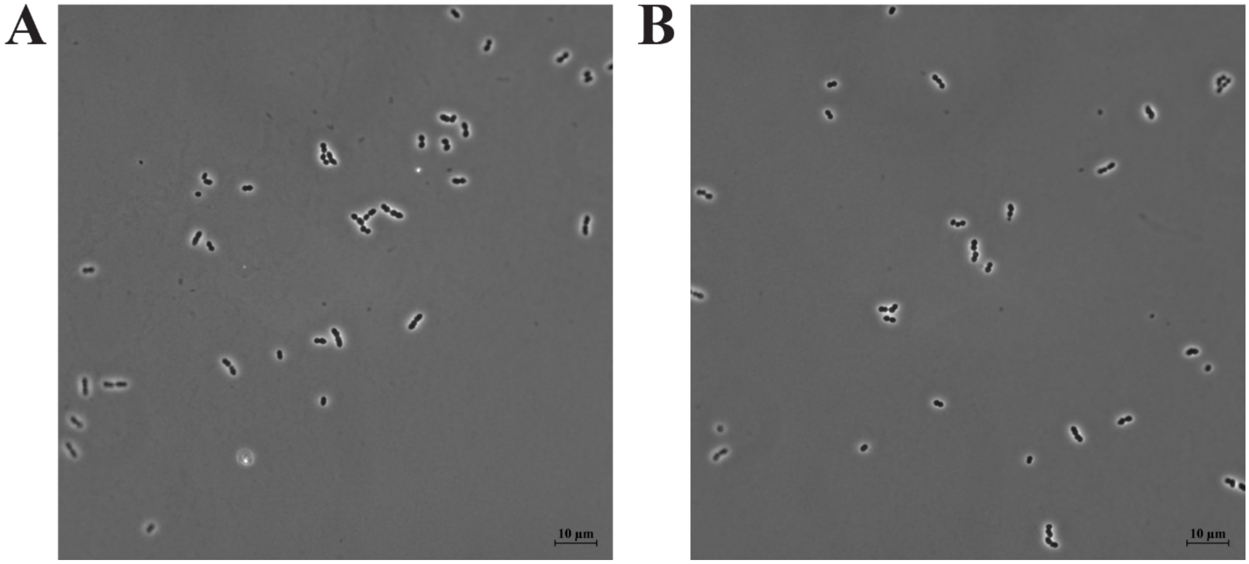

Fig S1. Phase contrast microscopy of *E. faecium* incubated for 2 hours in PBS (A), and with PBS containing 1 µM FITC-EntK1 (B).

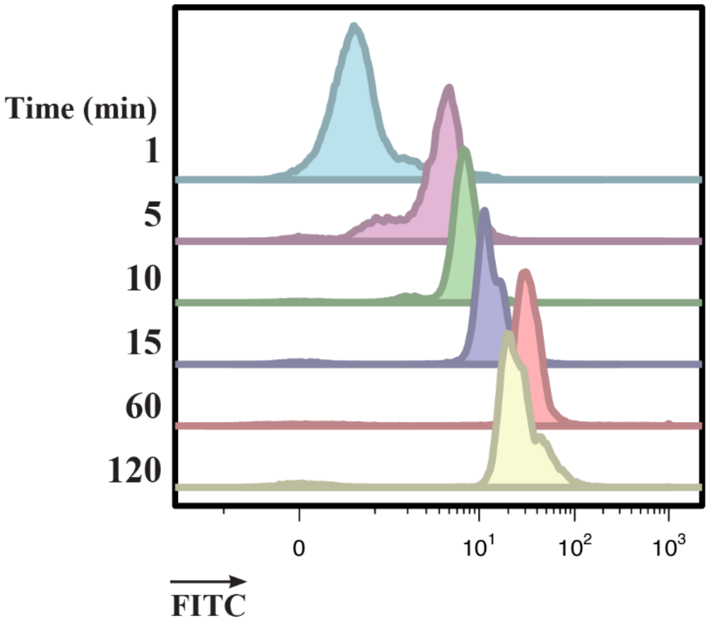

Fig S2. Effect of incubation time on the fluorescence intensity of *E. faecium* (10^5 CFU/ml). Cells were incubated in 0.1 mM triammonium citrate (pH 6.8) containing 0.2 μM FITC-EntK1 at various time points (1-120 minutes).

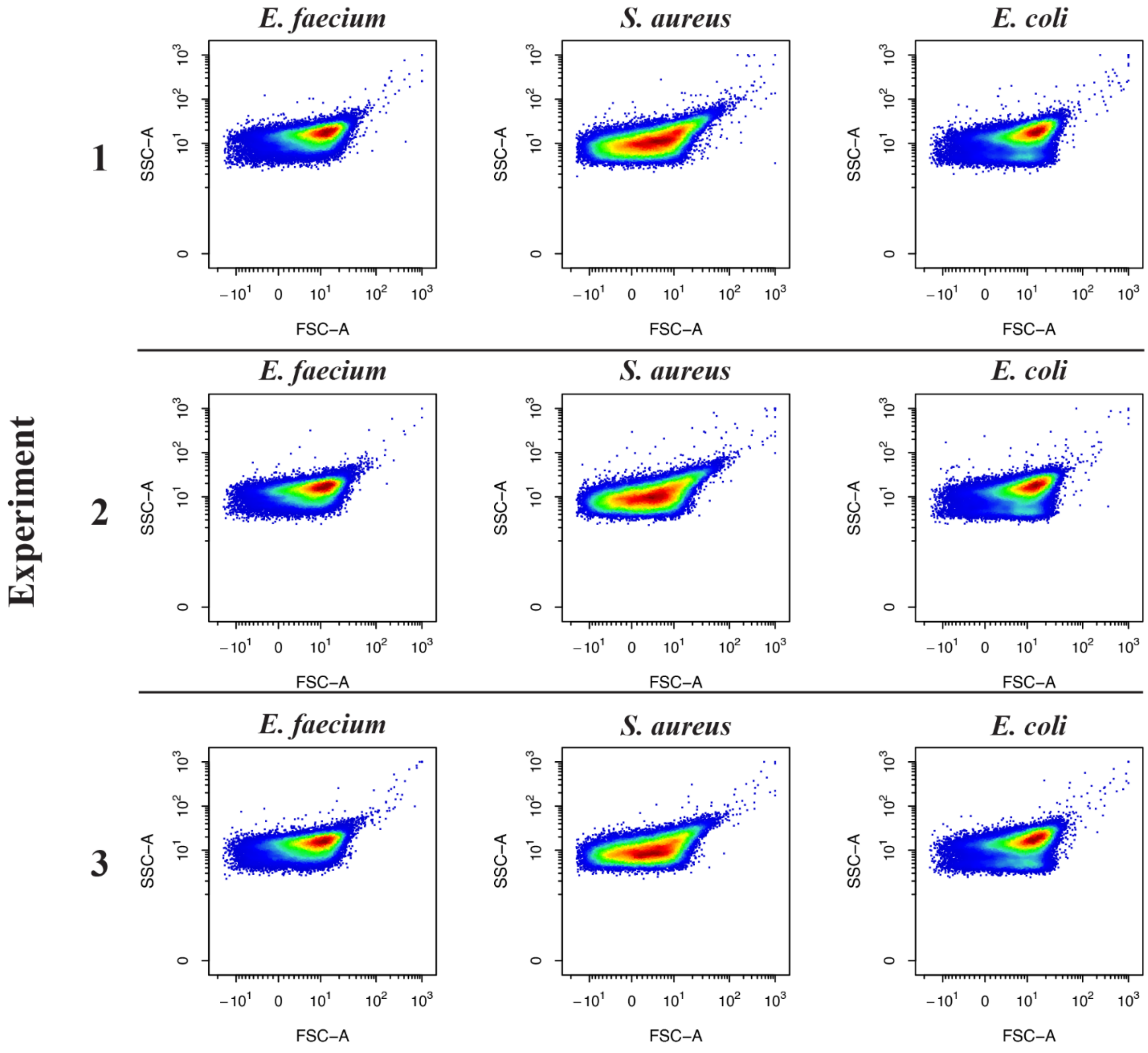

Fig S3. Density plots (dot plots) of flow cytometry measurements presented in Figure 5.

Table S1. Flow cytometry data obtained from the limit of detection experiment; values are representative from three biological replicates.

Cells (×10^3 CFU/ml)	Events	Gated (%)	MFI Gated
105	204431	130836 (64%)	19.4
58	121676	73234 (60%)	29.0
35	69371	37954 (55%)	32.0
22	44024	21157 (48%)	31.8
14	28003	10666 (38%)	31.9
5	21967	6355 (29%)	30.3
3	16809	3518 (21%)	30.1
0	12116	262 (2.2%)	0.11

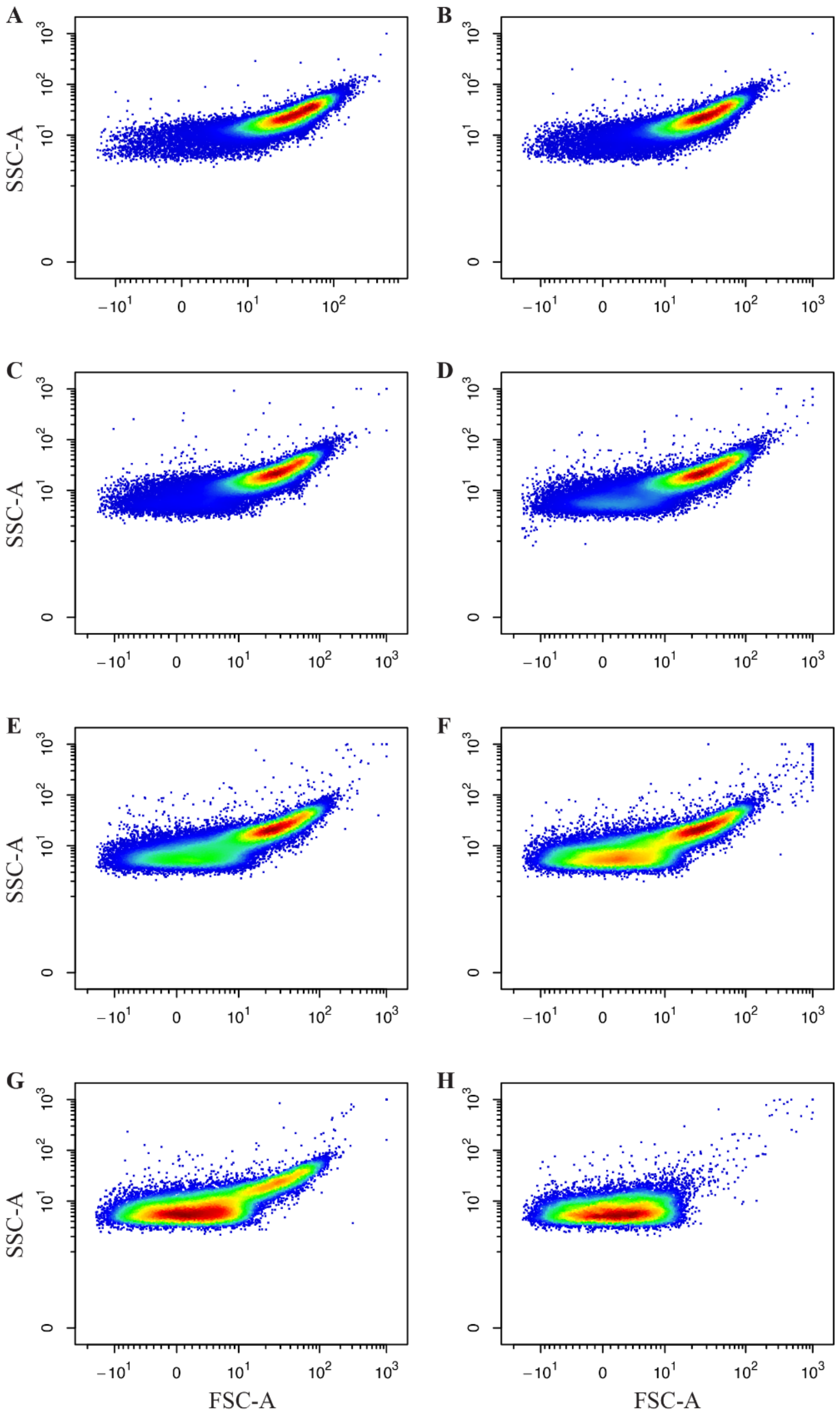

Fig S4. Representative density plots of the limit of detection experiment shown in Figure 7. Measurements were obtained from a urine sample inoculated with 105×10^3 (A), 58×10^3 (B), 35×10^3 (C), 22×10^3 (D), 14×10^3 (E), 5×10^3 (F), 3×10^3 (G), and 0 (H) CFU/ml (see Table S1).
